# Genome-wide identification of R2R3-MYB gene family and association with anthocyanin biosynthesis in *Brassica* species

**DOI:** 10.1186/s12864-022-08666-7

**Published:** 2022-06-14

**Authors:** Daozong Chen, Haidong Chen, Guoqiang Dai, Haimei Zhang, Yi Liu, Wenjie Shen, Bo Zhu, Cheng Cui, Chen Tan

**Affiliations:** 1 College of Life Sciences, Ganzhou Key Laboratory of Greenhouse Vegetable, Gannan Normal University, 341000 Ganzhou, China; 2 Crop Research Institute, Sichuan Academy of Agricultural Sciences, 610066 Chendu, China

**Keywords:** *Brassica* species, R2R3-MYB family, Subgenome, Anthocyanin biosynthesis, Co-expression

## Abstract

*Brassica* species include important oil crops and vegetables in the world. The R2R3-MYB gene participates in a variety of plant functions, including the activation or inhibition of anthocyanin biosynthesis. Although previous studies have reported its phylogenetic relationships, gene structures, and expression patterns in *Arabidopsis*, the number and sequence variation of this gene family in *Brassica* crops and its involvement in the natural quantitative variation in anthocyanin biosynthesis regulation are still largely unknown. In this study, by using whole genome sequences and comprehensive genome-wide comparative analysis among the six cultivated *Brassica* species, 2120 R2R3-MYB genes were identified in six *Brassica* species, in total These R2R3-MYB genes were phylogenetically clustered into 12 groups. The R2R3-MYB family between A and C subgenomes showed better collinearity than between B and C and between A and B. From comparing transcriptional changes of five *Brassica* species with the purple and green leaves for the detection of the R2R3-MYB genes associated with anthocyanin biosynthesis, 7 R2R3-MYB genes were co-differentially expressed. The promoter and structure analysis of these genes showed that some variations between non-coding region, but they were highly conserved at the protein level and spatial structure. Co-expression analysis of anthocyanin-related genes and R2R3-MYBs indicated that *MYB90* was strongly co-expressed with TT8, and they were co-expressed with structural genes *F3H*, *LDOX*, *ANS* and *UF3GT* at the same time. These results further clarified the roles of the R2R3-MYBs for leaf coloration in *Brasica* species, which provided new insights into the functions of the R2R3-MYB gene family in *Brasica* species.

**Supplementary Information:**

The online version contains supplementary material available at 10.1186/s12864-022-08666-7.

## Introduction

The roles of transcription factors (TFs) in the regulation of transcript levels of structural genes controls many crucial biological processes [2]. The TFs usually recognize target DNA in a sequence-specific manner and regulate the frequency of initiation of transcription upon binding to specific sites/motifs in the promoter of target genes. Generally, TFs can work as activators, repressors, or both to regulate the expression of target genes. MYB transcription factors have since been found in all plants and comprised one of the largest of transcription factors family [27]. MYB transcription factor contains a conserved DBD (DNA-binding domain), which is generally composed of 1–4 imperfect repeats, named R repeat [14, 27]. Each R repeat covers 50–55 amino acids in length that fold into three α-helices, when bound to target gene specific promoter sequences, the second and third α-helices were form a helix-turn-helix (HTH) structure [27, 31], and the third α-helix usually play a recognition role in binding to a short DNA sequence [43].

For decades, it has been identified and reported in many plants that the R2R3-MYB transcription factor is widely involved in plant development and metabolic regulation [13, 28, 43, 45–47]. Recently, the increasing availability of plant genome sequences have facilitated a better understanding of this large gene family [28, 45–47]. The first plant R3R3-MYB gene *COLORED1* (*C1*) was isolated from maize (*Zea mays*) which encode a regulatory protein involved in anthocyanin biosynthesis [32]. Subsequently, the R2R3-MYB-mediated anthocyanin synthesis pathway was revealed in the model plant *Arabidopsis*, *PRODUCTION OF ANTHOCYANIN PIGMENT 1/2* (*PAP1/2*, *MYB75*/*90*) are reported to be involved in the transcriptional regulation of anthocyanins in vegetative tissues, while *TRANSPARENT TESTA 2* (*TT2*, *MYB123*) is involved in the biosynthesis and accumulation of anthocyanins in seed coats [19, 52]. Nevertheless, compared to *Arabidopsis*, relatively few members of the *Brassica* species R2R3-MYB gene family have been well identified and functional characterized. Moreover, the R2R3-MYBs characterized in *Brassica* species are limited in the transcriptional regulatory mechanism of anthocyanin metabolism pathway to date.

Anthocyanins are secondary metabolites of flavonoids, which can change from red, purple to blue, and are the main substances for plant coloring in nature. Anthocyanins play an important role in plant growth and development, especially against biotic and abiotic stress. For humans, previous studies have found that anthocyanins can scavenge free oxygen, improve vision, delay aging and used in cancer treatment. In recent years, R2R3-MYB transcription factors have been shown to be involved in phenylpropanoid metabolism [23, 41] and activate the structural genes in the anthocyanin biosynthetic pathway in many plants [1, 20, 51]. In *Brassica* species, the R2R3-MYB transcription factors play a key role in anthocyanin biosynthesis pathway. In *B. oleracea*, *BoMYB2* with different types of mutations in the promoter region determines the different color types [53]. Similarly, the activated expression of the *BrMYB2* gene in purple-leaf *B. rapa* and the *BjPur* gene in purple-leaf *B. juncea* is due to the large deletion of the first intron [21, 22]. In *B. napus*, *BnaPAP2.A7* has been found to work as a key transcription factor regulating the synthesis of anthocyanins in the leaves [8]. However, both *BoMYB2*, *BrMYB2*, *BjPur* and *BnaPAP2.A7* are homologous of *Arabidopsis AtPAP2*, while other R2R3-MYB transcription factors regulate the synthesis of anthocyanins in *Brassica* crops are rarely need to be explored.

In U’s Triangle *Brassica* species, *B.rapa*, *B.oleracea* and *B.nigra* genomes (designated A, B and C) share mesohexapolyploid ancestry and occur both singly and in each pairwise combination to define the *Brassica* species, which is the excellent model for studying the evolution of gene families. In this study, we performed genome-wide identification of MYB transcription factors of the six cultivated *Brassica* species. R2R3-MYB and R1R2R3-MYB subfamilies were identified and analyzed, including chromosomal distribution, synteny and evolutionary relationships. We also analyzed the spatial and temporal expression profiles as well as differential expression profiles of R2R3-MYB genes in five *Brassica* species (except *B. nigra*) with green and purple leaves, and then the gene structure, conserved domains and original promoters. These findings will provide a comprehensive understanding of novel R2R3-MYB genes involved in anthocyanin biosynthesis and assist further exploration of the functional characteristics of R2R3-MYB transcription factors in *Brassica* species.

## Materials and methods

### Identification of R2R3-MYB transcription factors in *Brassica*  

In this study, the genome and protein sequences of the *B. rapa* (Chiifu), *B. oleracea* (TO1000), *B. nigra* (Ni100), *B. napus* (ZS11) were downloaded from the BRAD database ([9, 10]; http://brassicadb.cn), *B. juncea* (SY) genome sequence from NCBI PRJNA615316 (Kang et al., 2021; https://www.ncbi.nlm.nih.gov/), *B. carinata* (zd-1) genome sequence from GenBank JAAMPC000000000 (Song et al., 2021; https://www.ncbi.nlm.nih.gov/), and the corresponding R2R3-MYB genome and protein sequences from the *Arabidopsis* database (TAIR; http://www.Arabidopsis.org/).

The candidate R2R3-MYB members in the six *Brassica* species were identified by a local BLASTP search with 125 R2R3-MYB genes from *Arabidopsis* to identify candidates with E-value < 1e-10, then all putative R2R3-MYB genes were identified in the PFAM protein family database (https://pfam.xfam.org/) using the HMMER software version 3.0 [15] and using the hidden Markov model of the MYB-DNA-binding domain (PF00249) and Myb_DNA-bind_6 domain (PF13921) to search against the six *Brassica* species genome to identify candidates with E-value < 1e-10 [17]. All protein sequences were further investigated using different online tools, including the motif search (https://www.genome.jp/tools/motif/), SMART (http://smart.embl-heidelberg.de/), ScanProsite (https://prosite.expasy.org/scanprosite/), NCBI-Conserved Domains Database (CDD) web server (https://www.ncbi.nlm.nih.gov/Structure/cdd/cdd.shtml), MEME Suite (https://meme-suite.org/meme/doc/meme-format.html) [28, 45].

### Phylogenetic analysis

The R2R3-MYB protein sequences of the six *Brassica* species and *Arabidopsis* were used to generate phylogenetic trees via ClustalX [26] and MAFFT sofaware [24] multiple sequence alignments with the default parameters. A maximum likelihood (ML) phylogenetic tree was constructed using FastTree2 software (v2.1.11), in which JTT (Jones-Taylor-Thornton) model was the best substitution model [34]. Additionally, itol (https://itol.embl.de/) and Adobe Illustrator CS6 software (v16.0.0) were used to modify the evolutionary tree.

### Chromosomal location and synteny analysis

The genome annotation data were collected and mapped on the chromosomes using the TBtools software (v0.67) to identify the physical chromosomal location of all R2R3-MYB genes in six *Brassica* crops and *Arabidopsis* [7]. The collinearity of intraspecific and interspecific genes was determined using the BLASTP (E-value: 1e-10, max_target_seqs:1) and Multiple Collinearity Scan toolkit (MCSscanX, gap_penalty: -1, E-value: 1e-10) [49], TBtools software (v0.67) was used to drop the collinearity genes on each chromosome [7].

### Expression profiles analysis based on RNA-seq data

The raw data of RNA-seq were downloaded from NCBI SRA database with item number PRJNA298501 (*B.rapa*, *B.oleracea*, *B.napus*, *B.juncea*, *B.carinata*, green and purple leaves), PRJNA430791, PRJNA359160 (*B.rapa*, green and purple leaves), PRJNA474411 (*B.oleracea*, green and purple curds), PRJNA558197 (*B.rapa*, green and purple leaves), PRJNA312129 (*B.rapa*, green and purple leaves) and PRJNA560282 (*B.oleracea*, green and purple leaves). And 144 seed coats RNA-seq data of six *Brassica* species (*B.rapa*, *B.oleracea*, *B.nigra*, *B.napus*, *B.juncea*, *B.carinata*) were collected from Gene Expression Omnibus under accession no.GSE153257. Low-quality reads were removed from the raw reads using Cutadapt and Trimmomatic software to get clean reads [4, 29]. Clean reads were mapped to the corresponding reference genome using HISAT2 software [33]. Gene expression levels of each gene were calculated using StringTie and Ballgown software [33]. The read counts of each gene were calculated using the htseq-count function in htseq software [3]. The R package DEseq2 (v1.16.1) was used to identify the differentially expressed genes (DEGs) between leaves of different colors based on the following criteria: padj < 0.05 & log2FoldChange > 2 [8].

### Analysis of the promoter characteristics, gene structure, conserved motif and co-differentially expression of R2R3-MYBs

Five groups green and purple leaves RNA-seq data were used to co-differentially expression R2R3-MYBs analyze, they are *B.rapa* (PRJNA359160), *B.oleracea* (PRJNA474411), *B.napus* (PRJNA298501), *B.juncea* (PRJNA430791), *B.carinata* (PRJNA298501). The co-differentially expression R2R3-MYB promoter regions of 2000 bp regions upstream of the translational start sites ATG were examined based on their positions in the genomes of six *Brassica* species and *Arabidopsis* using Samtools software (v 1.8), which was used to identify the cis-elements in the promoters according to the online PlantCARE database (http://bioinformatics.psb.ugent.be/webtools/plantcare/html/). The gene structures of all co-differentially expression R2R3-MYB genes were analyzed according to the GFF annotation file of the gene position information in the six *Brassica* plants and *Arabidopsis* database. TBtools software (v0.67) was used to locate the co-differentially expression R2R3-MYB to the different chromosomes of each species, including exon and intron numbers and lengths [7]. The MEME online tool (https://meme-suite.org/meme/) was used to investigate conserved domains, and the WEBLoGo online tool (https://weblogo.berkeley.edu/) and AlphaFold Protein Structure Database (https://www.alphafold.ebi.ac.uk/) were used to analyze the R2 and R3 motif conserved sequence and SWISS-MODEL online tool (https://swissmodel.expasy.org/) was used to draw spatial structure. The RNA-seq data were used to perform co-expression network analysis using R language (v3.6.1). In order to calculate the adjacent order function formed by the gene network and the difference coefficients of different nodes, the TOM similarity algorithm calculates the co-expression correlation matrix to express the gene correlation in the network. The correlation network diagram was drawn by extracting the non-weight coefficients (weight) of R2R3-MYB and anthocyanin-related genes in the matrix. STRING software (https://version-11-5.string-db.org/) was used to reveal a co-expression plot [28, 45].

### Plant materials, RNA extraction, and qRT-PCR analysis

The *B. rapa*, *B. oleracea*, *B. napus*, *B. juncea* and *B. carinata* green and purple leaves of seven-leaf stage with three biological replicates were collected, all samples were collected and immediately frozen in liquid nitrogen for RNA extraction and qRT-PCR analysis. Six genes (*BraA07g032100.3 C*, *Bo3g081880*, *Bo6g100940*, *BnaA07T0287000ZS*, *BnaC06T0329100ZS* and *BcaB05g24263*) have been used the primer (RTPAPA7-3-F: GCATTGATAAGTATGGAGAAGG, RTPAPA7-744-R3: GACCACCTGTTTCCTAAAAGC) for qRT-PCR analysis, the primer (Bju-PAP2-qF: ATGGATGATCTGTCGTATAGG, Bju-PAP2-qR: CAGCTCTTGCAGGAACTA) was used to qRT-PCR analysis for *BjuB05g10740S*, and the primer (Bca-PAP2C3-qF: CCTGGACTCAATGACACTA, Bca-PAP2C3-qR: AACCTCCGCGTTTGACGT) was used to qRT-PCR analysis for *BcaB03g15272*. The Actin3 gene (Bnaactin3-qF: TCCATCCATCGTCCACAG, Bnaactin3-qR: GCATCATCACAAGCATCCTT) as an internal reference gene control. For detailed methods, please refer to Chen et al. [8]. All materials of this research were collected from Gannan Normal University research base.

## Results

### Identification and characterization of R2R3-MYB family genes

The 125 R2R3-MYB genes protein sequences of *Arabidopsis* were downloaded and used as seed sequences to search the protein sequences database of six *Brassica* plants and *Arabidopsis* to identify homologous R2R3-MYB genes in six *Brassica* plants. Firstly, the candidate R2R3-type MYB members in the six *Brassica* plants were identified by a local BLASTP search with *Arabidopsis* to identify candidates with E-value < 1e-10. Secondly, Hidden Markov model (HMM) profile of the MYB domain Myb-DNA-binding domain (PF00249) and Myb_DNA-bind_6 domain (PF13921) queried the hmmsearch program (HMMER3.0 package) against the protein database of six *Brassica* plants and *Arabidopsis*. Subsequently, all protein sequences were further investigated using hmmscan, SMART, ScanProsite, NCBI-CDD search and MEME Suite. A total of 2472 MYB genes were identified, these included 297 1R-MYB genes, 2120 R2R3-MYB genes, 33 R1R2R3-MYB genes, and 22 R1R2R3R4-MYB genes (Table 1, Supplementary Table S1). Among them, the R2R3-MYBs were the largest MYB subgroup containing 2120 genes, including 130 in *Arabidopsis*, 236 in *B. rapa*, 247 in *B.oleracea*, 248 in *B. nigra*, 425 in *B. napus*, 422 in *B. juncea* and 412 in *B. carinata* (Table 1).

**Table 1:** R2R3_MYB protein distribution in six *Brassica* crops

species	R2R3_MYB	3R_MYB	>=4R_MYB	R1_MYB	Total
*A.thaliana*	130	2	1	23	156
*B.carinata*	412	7	3	48	470
*B.juncea*	422	5	5	57	489
*B.napus*	425	9	4	63	501
*B.nigra*	248	2	3	36	289
*B.oleracea*	247	4	4	41	296
*B.rapa*	236	4	2	29	271
total	2120	33	22	297	2472

### Phylogenetic analysis of R2R3-MYB genes in six *Brassica* species

**Fig. 1:**
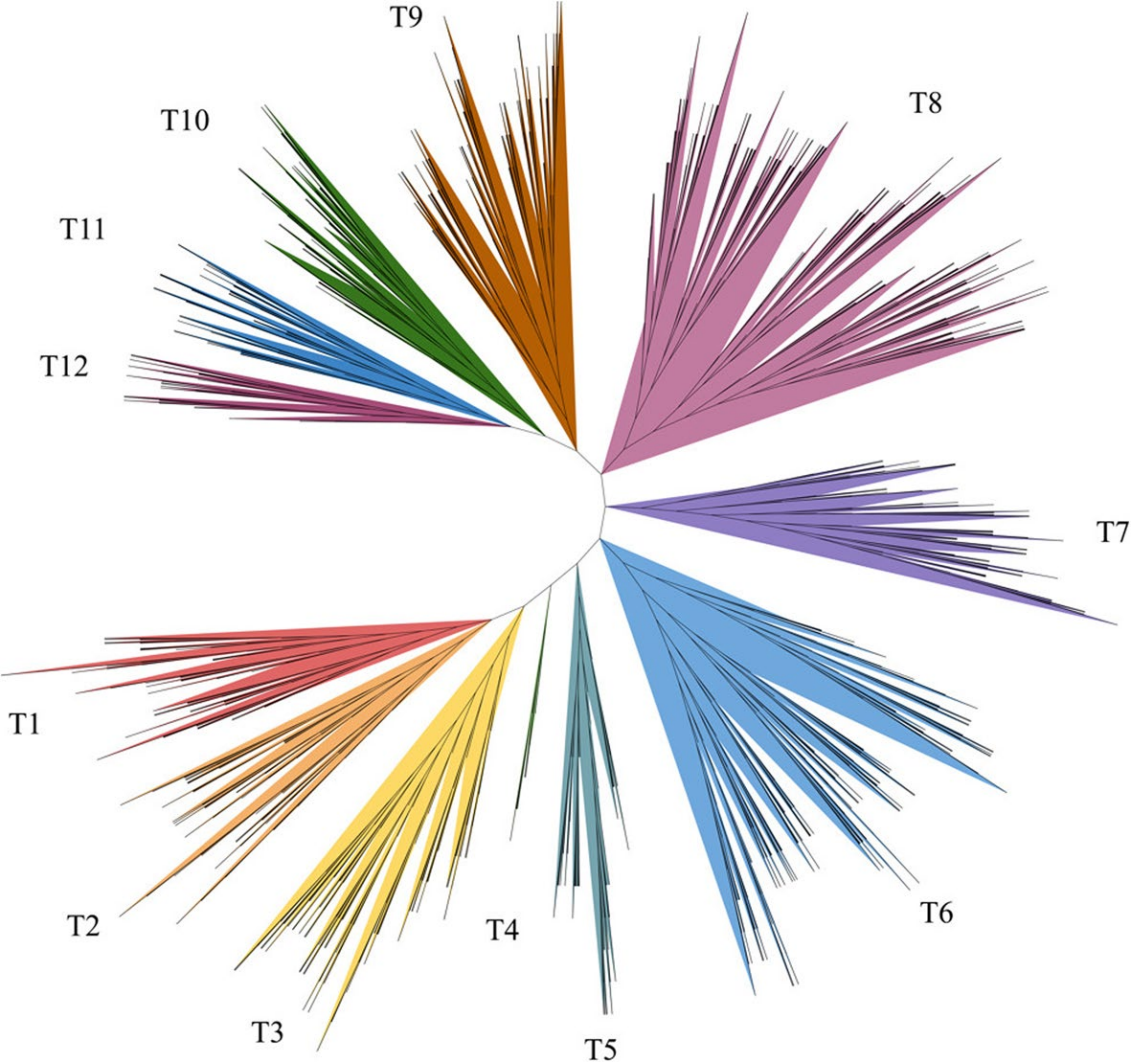
Phylogenetic relationships of R2R3-MYB proteins between six Brassica crops and Arabidopsis. The lines represented genes from six Brassica crops and Arabidopsis, respectively. All 12 subfamilies of R2R3-MYBs were well separated in different clades and represented by different colors. The ML phylogenetic tree was generated using JTT algorithm with 1,000 bootstrap value via FastTree2

To analyze the phylogenetic relationships and gene functions of the R2R3-MYB gene family members, a ML tree containing 2120 R2R3-MYB genes was constructed using mafft software for multiple sequence alignment of protein sequences, and using fasttree software a clustering tree was built. The 2120 R2R3-MYB genes could be divided into 12 subfamilies (T1-T12) and were drawn in different colors (Fig. 1). Compared with *Arabidopsis* 25 subfamilies, our T1 was the S21 subfamily, T2 contained the S22 and S23 subfamilies, T3 was the S25 subfamily, T5 contained the S6 and S15 subfamilies, T6 contained the S8, S18, S19 and S20 subfamilies, T7 contained the S13 and S16 subfamilies, T8 contained the S1, S2, S3, S5 and S12 subfamilies, T9 contained the S9, S10, S11 and S24 subfamilies, T10 was the S14 subfamily, T11 included the S4 and S7 subfamilies, T12 was the S17 subfamily (Supplementary Table S2). T4 subfamily contained one R2R3-MYB gene in *Arabidopsis* and 18 homologous in *Brassica* plants, which was separated into one subfamily (Supplementary Table S3). Previous research has shown that the group of R2R3-MYB genes in the same subclade mighty have a similar function [43, 52]. The R2R3-MYB gene functions in the T5, T8 and T11 subgroups were known to be involved in the phenylalanine metabolism pathway, including the regulation of anthocyanin and procyanidin synthesis [14]. For example, in T11 subgroup, *AT1G22640* (*AtMYB3*), *AT4G38620* (*AtMYB4*), *AT2G16720* (*AtMYB7*), and *AT4G34990* (*AtMYB32*) inhibited phenylalanine biosynthesis; AT2G47460 (*AtMYB11*), *AT3G62610* (*AtMYB12*) and *AT5G49330* (*AtMYB111*) had the ability to participate the PA biosynthesis. The T11 subfamily contained *AT1G56650* (*AtMYB75*), *AT1G66390* (*AtMYB90*), *AT1G66370* (*AtMYB113*), and *AT1G66380* (*AtMYB114*) that regulated anthocyanin biosynthesis in vegetative tissues [52].

### Chromosomal distribution and collinearity analysis of R2R3-MYBs in six *Brassica* species

All 2120 2R3-MYB genes were mapped onto the corresponding genomic chromosomes and were found to be located onto all chromosomes of each species, i.e., 130 genes in *Arabidopsis* to 5 chromosomes, 236 genes in *B. rapa* to 10 chromosomes, 247 genes in *B. oleracea* to 9 chromosomes, 248 genes in *B. nigra* to 8 chromosomes, 425 genes in *B. napus* to 19 chromosomes, 422 genes in *B. juncea* to 18 chromosomes and 412 genes in *B. carinata* to 17 chromosomes (Supplementary Fig. 1). The results showed that the R2R3-MYB gene was relatively evenly distributed in each subgenome of the six *Brassica* crops, and only relatively few in the B subgenome of *B.carinata*, with only 166 members (Table 2). Among the three subgenomes of A, B, and C, the A subgenome has the most distribution (Supplementary Fig. 1).

**Table 2:** Distribution of R2R3_MYB gene family in each subfamily

species	AT	B.ra	B.ol	B.ni	B.ju	B.na	B.ca	Total
**T1**	8	16	17	18	31	35	29	154
**T2**	8	14	16	15	28	32	24	137
**T3**	12	21	24	21	39	37	32	186
**T4**	1	2	2	2	4	4	4	19
**T5**	8	10	10	13	21	18	21	101
**T6**	23	40	39	39	70	76	67	354
**T7**	12	22	21	23	38	37	34	187
**T8**	22	48	52	52	90	81	93	438
**T9**	13	27	27	27	38	43	43	218
**T10**	8	15	16	15	29	23	24	130
**T11**	9	12	12	14	21	21	25	114
**T12**	6	9	11	8	13	18	17	82
**all**	130	236	247	247	422	425	413	2120

**Fig. 2:**
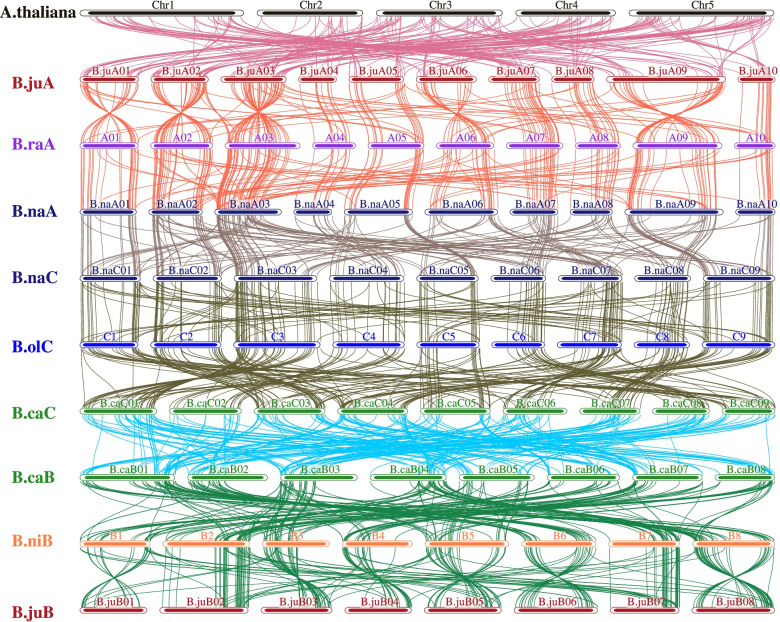
Interspecies synteny of R2R3-MYBs between the six species in U’s triangle and Arabidopsi. R2R3-MYBs colinearity of the genomes of the six species in U’s triangle and Arabidopsis, including three diploid species, B. rapa (A genome, BraA), B. nigra (B genome, BniB), and B. oleracea (C genome, BolC) and three tetraploid species, B. napus (AACC, BnaA, and BnaC subgenomes); B. juncea (AABB, BjuA and BjuB subgenomes); and B. carinata (BBCC, BcaB and BcaC subgenomes)

The comparison of interspecific synteny among six *Brassica* species and *Arabidopsis* were also analyzed to further explore the evolution of R2R3-MYB genes (Fig. 2). At the genome-wide level, three A subgenomes from *B. rapa*_(BraA), *B. napus*_(BnaA), *B. juncea*_(BjuA) had a good collinearity relationship, almost all collinearity blocks were distributed on the corresponding homologous chromosomes of the same number, showing that the A subgenome had the less degree of gene differentiation and better preservation of gene integrity during the evolution process (Fig. 2). In three C subgenomes from *B. oleracea*_(BolC), *B. napus*_(BnaC) and *B. carinata*_(BcaC), BolC and BnaC shared a good collinearity relationship, and all collinearity blocks were distributed in the corresponding homologous chromosomes of the same number. However, the chromosomal correspondence between the subgenome BnaC and BcaC was poor, which indicated that the C subgenome had a less degree of gene differentiation and better preservation of gene integrity during the evolution of *B. napus*, while the chromosomal level variation was higher in *B. carinata*. Similarly, in the three B subgenomes from *B. nigra*_(BniB), *B. juncea*_(BjuB) and *B. carinata*_(BcaB), BniB and BjuB gave a good collinearity relationship, and most of the collinearity blocks were distributed onto the corresponding same numbers on homologous chromosomes. However, the chromosomal correspondence between BniB and BcaB subgenome was poor, indicating that the B subgenome had a lower degree of gene differentiation and better preservation of gene integrity during the evolution of *B. juncea*, while the *B. carinata* had a larger chromosome level variation (Fig. 2).

### Analysis of the transcriptome and co-differentially expressed R2R3-MYBs

**Fig. 3:**
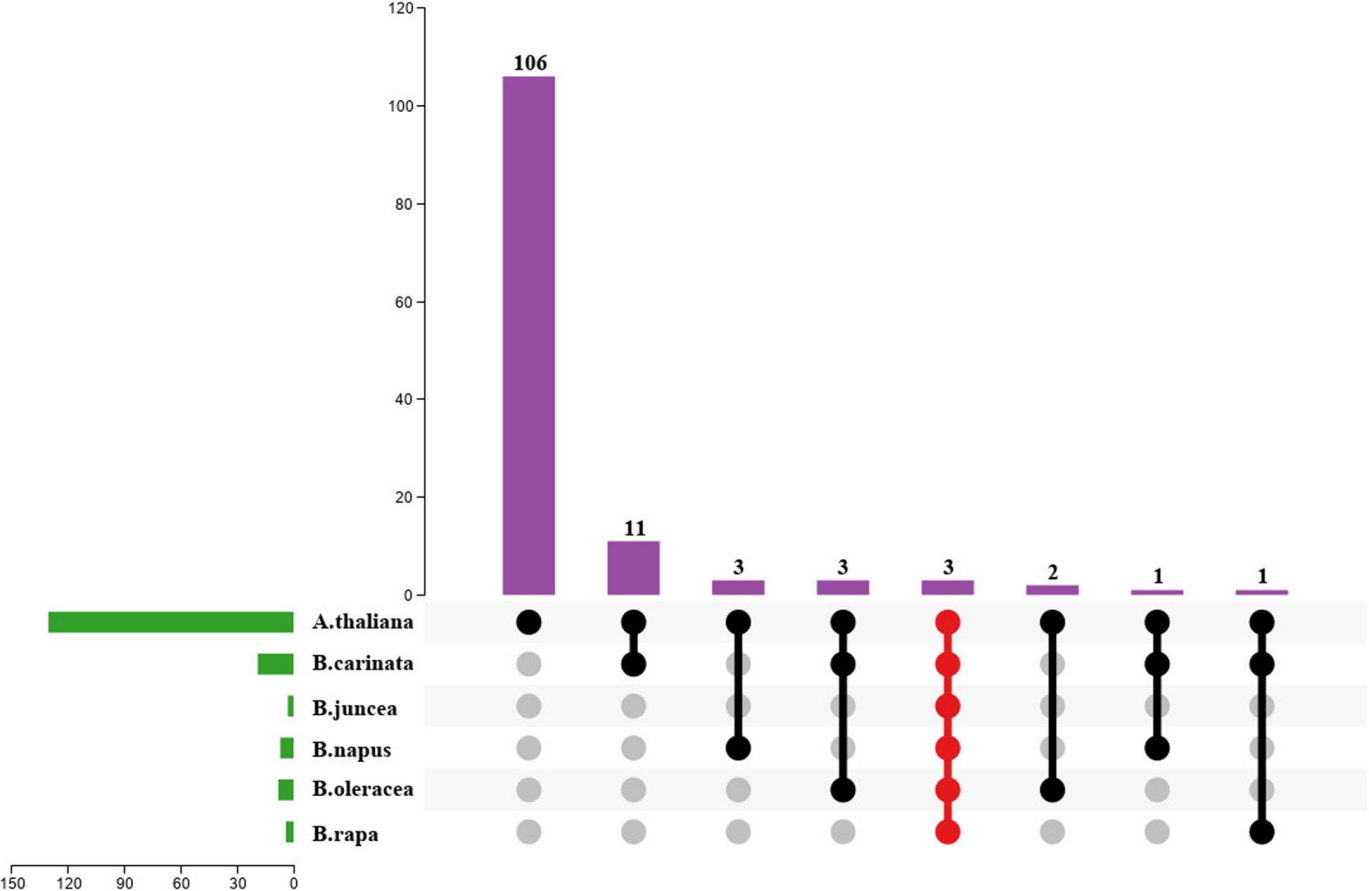
The differentially expressed R2R3-MYBs between five Brassica crops of green and purple leaves RNA-seq data. The red color indicates the co-differentially expressed R2R3-MYBs in five Brassica crops

The different tissues with green, white or purple phenotype of *B. rapa*, *B. oleracea*, *B. napus*, *B. juncea* and *B. carinata* RNA-seq data were used to determine expression levels of R2R3-MYBs. Compared to the genome-wide analysis of the R2R3-MYB gene family, 41 R2R3-MYBs were found to be differentially expressed in transcriptome data. All these differentially expressed R2R3-MYBs (DE R2R3-MYBs) exhibited differential expressions between green and purple leaves. There were 9, 6, 11, 8, 7 DE R2R3-MYBs in *B. rapa*, *B. oleracea*, *B. napus*, *B. juncea* and *B. carinata*, respectively (Supplementary Table S4). Among them, 7 genes were differentially expressed in common, which were all homologous copies of *Arabidopsis AT1G56650* (*MYB75*), *AT1G66370* (*MYB113*), *AT1G66380* (*MYB114*) and *AT1G66390* (*MYB90*) genes (subfamily 6 of R2R3-MYBs). In five *Brassica* crops, they were *BraA07g032100.3 C*, *Bo3g081880*, *Bo6g100940*, *BnaA07T0287000ZS*, *BjuB05g10740S*, *BcaB05g24263*, *BcaB03g15272* (Supplementary Table S4). In *B. napus*, the DE R2R3-MYBs *BnaA07T0287000ZS* and *BnaC06T0329100ZS* were all up-regulated in the purple leaf. Interestingly, of two differentially expressed genes in *B. carinata*, *BcaB05g24263* was down-regulated in purple leaves, while *BcaB03g15272* was up-regulated (Fig. 3).

### Gene structural analysis and conserved motif identification of DE R2R3-MYBs

Gene structural analysis was helpful for the better understanding of its function and evolution. The intron numbers of the 7 DE R2R3-MYBs and *Arabidopsis AT1G56650* (*MYB75*), *AT1G66370* (*MYB113*), *AT1G66380* (*MYB114*) and *AT1G66390* (*MYB90*) were all three, except for four introns of *Bo3g081880* (Supplementary Fig. 2). However, there were large variations in the length of exons and introns, especially for *BraA07g032100.3 C* with a large insert in the first intron.

The 2,000 base pairs (bp) upstream of R2R3-MYB gene sequences of the coding region were used to predict *cis* regulatory elements via the PlantCARE online tool (Supplementary Fig. 2). A total of 20 *cis*-regulatory elements of DE R2R3-MYBs were predicted, including 3 cellular development related *cis*-regulatory elements: cell cycle regulation, meristem expression, maximal elicitor-mediated activation, 5 hormone related *cis*-regulatory elements: abscisic acid responsiveness, auxin responsive, MeJA- responsiveness, gibberellin responsiveness, salicylic acid responsiveness. Similarly, 4 stress related *cis*-elements were also identified, including light responsiveness, defense and stress responsiveness, low-temperature responsiveness, dehydration, low-temp, salt stress. The MBS and MRE were specifically MYB binding sites involved in flavonoid biosynthetic, light responsiveness and drought-inducibility. AT-rich DNA binding protein, core promoter element around − 30, MYBHv1 binding, protein binding site and maximal elicitor-mediated activation. The numbers of *cis*-regulatory elements for each DE R2R3-MYBs varied, *Bo6g100940* had only one and *BcaB05g24263* had nine.

All DE R2R3-MYBs contained two highly conserved MYB binding domains. The motif logo of DE R2R3-MYBs had 51 amino acid residues in the R2 repeat and 45 amino acid residues in the R3 repeat, respectively (Supplementary Fig. 3). The HTH structure of these two domains as revealed by three-dimensional (3D) protein structural models showed that DE R2R3-MYBs matched the typical characteristics of the R2R3-MYB family (Supplementary Fig. 3).

### Co-expression analysis of anthocyanin-related genes and R2R3-MYBs

**Fig. 4:**
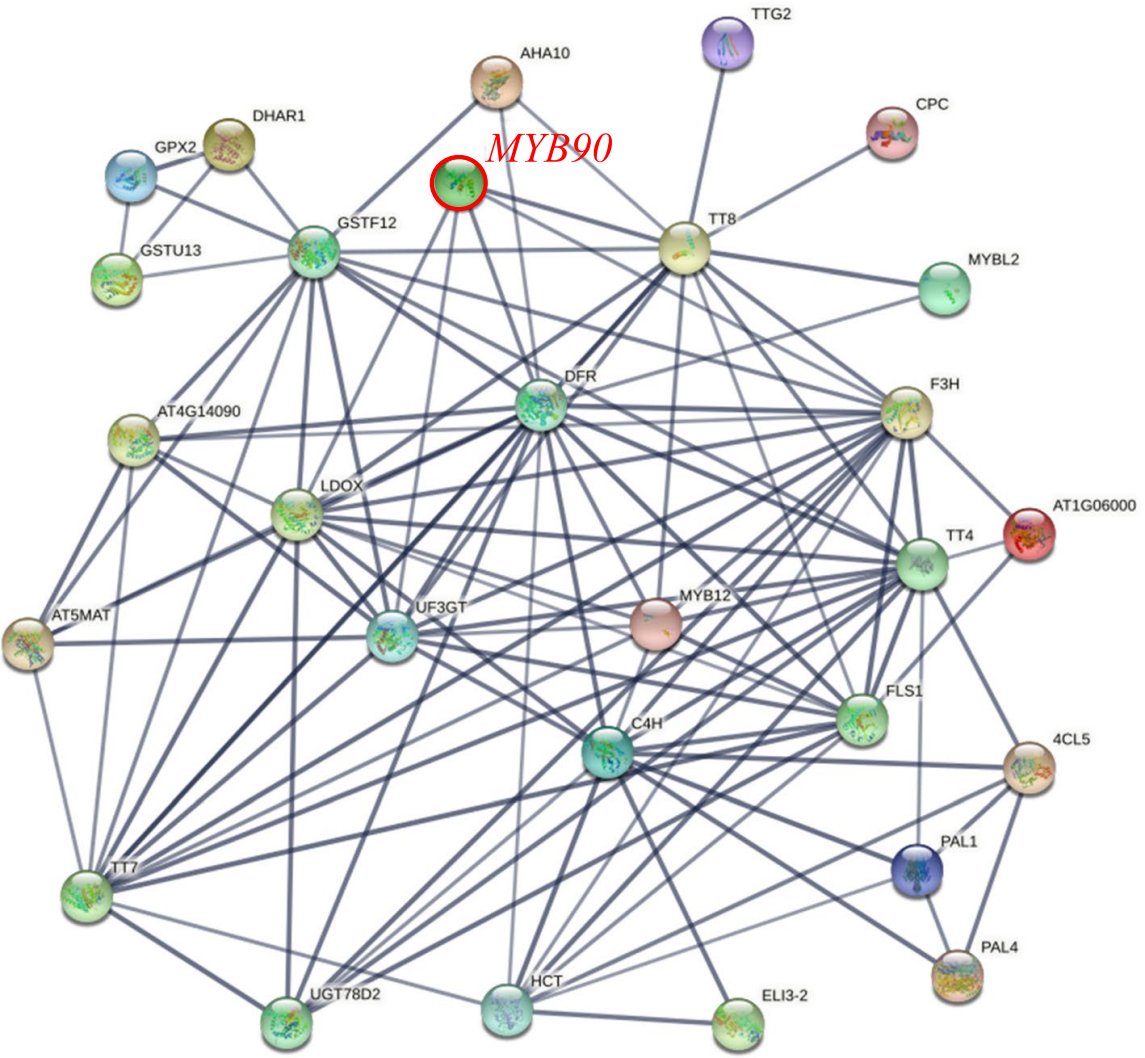
Co-expression networks of R2R3-MYB with anthocyanin biosynthesis pathway genes of five Brassica species. The connecting line indicates that there is interaction between each other, and the thickness of the line represents the strength of the interaction

The DEGs between the green, white or purple tissues of five *Brassica* species were used to predict candidate R2R3-MYBs related to anthocyanin biosynthesis (Supplementary Table S4). *MYB90* (representing *MYB75*, *MYB90*, *MYB113*, *MYB114* and eight DE R2R3-MYBs in *Brassica* plants) had a strong co-expression with *TT8*, and they were co-expressed with structural genes *F3H*, *LDOX, ANS* and *UF3GT* at the same time (Fig. 4). Simultaneously, the anthocyanin biosynthesis repressor *CPC* and *MYBL2* were also co-expressed with *TT8*. These results showed that these eight DE R2R3-MYBs were the important regulation genes involved in anthocyanin biosynthesis processes.

### Expression pattern of R2R3-MYB DEGs and anthocyanin related genes in five *Brassica* species

**Fig. 5:**
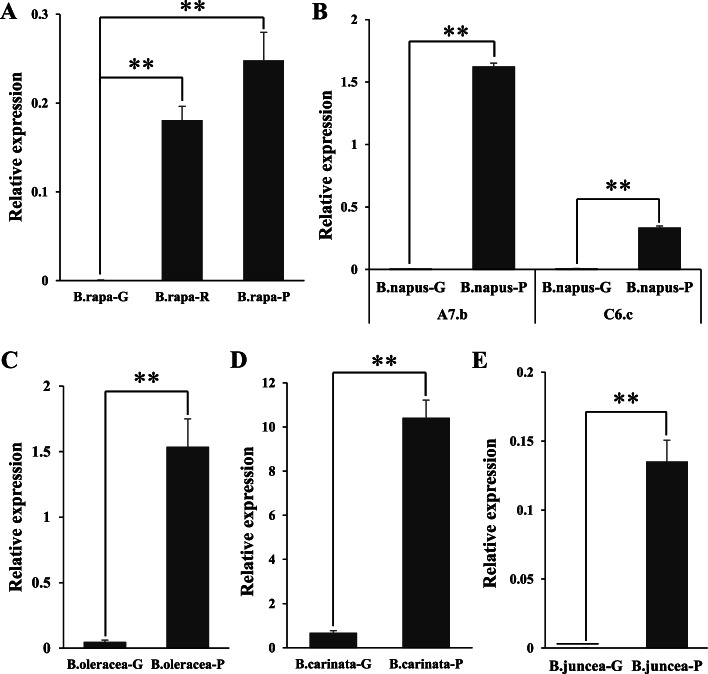
qRT-PCR analysis of AtPAP2 homologous relative expression in five Brassica species with green and purple leaves. For qRT-PCR analysis, both five Brassica species green and purple samples are with three biological replicates, the point represents the mean value of three technical replicates in a representative biological experiment, the error bars indicate s.d, student’s t-test, **P<0.01,*P<0.05

In order to confirm the transcriptional pattern revealed by the RNA-seq analysis, we performed qRT-PCR analysis for 7 DE R2R3-MYBs (Fig. 5). Six genes showed a similar relative expression level in green / white and purple tissues as revealed by RNA-seq analysis. The *MYB90* was hardly expressed in the green / white tissues of five *Brassica* species, but significantly upregulated in the purple/red tissues, which was consistent with the transcriptome and phenotype, respectively. However, *BcaB03g15272* is not expressed in purple and green leaves, which is different from the results of the transcriptome.

## Discussion

### The R2R3-MYB gene family in *Brassica* species

The R2R3-MYB is the largest subfamily of the MYB transcription factor family. To date, several R2R3-MYB families have been identified and analyzed. In the model plant *(A) thaliana*, 125 R2R3-MYB genes were found [43], and then 126 R2R3-MYB genes detected from the whole genome MYB transcription factor identification and divided into 26 subfamilies [14], which provides a reference for the identification and analysis of R2R3-MYB transcription factors in other plants. In recent years, numerous R2R3-MYB transcription factors have been identified and functionally analyzed, such as in *Oryza sativa* [11], *Zea mays* [13], *(B) napus* [20] and horticultural plants *Petunia* [9, 10], Strawberry [28] and *Capsicum annuum* [45]. Importantly, more and more biological pathways involved in R2R3-MYB have been revealed, such as the transcriptional regulation of anthocyanins [56], fiber development [45] and root hair development [42].

Six *Brassica* species of three diploids and three derived allotetrploids in U’s triangle are excellent models for studying gene families. With the completion of the sequencing of the *B. carinata* genome, the genomes of all six species have been published, providing a useful tool for genome-wide analysis of the R2R3-MYB gene family. To date, no full analysis of the R2R3-MYB gene family is reported in these six *Brassica* crops and most functions remain unclear. In this study, we identified 2120 R2R3-MYB genes from the genomes of *Arabidopsis* and six *Brassica* crops, including 130 in *Arabidopsis*, 236 in *B. rapa*, 247 in *B. oleracea*, 248 in *B. nigra*, 425 in *B. napus*, 422 in *B. juncea* and 412 in *B. carinata* (Table 1), indicating the expansion of this gene family along with genome duplication in *Brassica* crops. The present numbers of R2R3-MYBs in *Arabidopsis*, *B. rapa* and *B. napus* were different from those of previous studies, 126 in *Arabidopsis* [43], 256 in the *B. rapa* [48], and 249 in the *B. napus* [20]. The main reason was probably that with the development of three-generation sequencing, genome information became more complete.

### Evolution of the R2R3-MYB gene family

Gene duplication is a major factor responsible for the expansion of gene families and the generation of new genes, which has also led to changes in the number and function of duplicated genes, and also provides an excellent example for studying the inheritance and evolution of gene family. The six *Brassica* crops of U’s triangle provides an excellent model for gene duplication and evolution research, because of the natural cross between the two diploids and the independent evolution of the parents and the allopolyploidy hybrids. To understand the evolutionary relationship of R2R3-MYB family genes in U’s Triangle six *Brassica* species, we constructed the phylogenetic tree with R2R3-MYB family genes from the U’s Triangle six *Brassica* species and *Arabidopsis*. All 2120 R2R3-MYB family genes from *Arabidopsis* and six *Brassica* crops were classified into 12 different classes with 130 R2R3-MYB family genes from *Arabidopsis* (Fig. 1), each cluster corresponds to one or more R2R3-MYB subfamily of *Arabidopsis* (Supplementary Table S3), which was in accordance with that the R2R3-MYB family was relatively conservative between different plants in evolution. Chromosomal distributions indicated that they were relatively evenly distributed on the A, B, and C subgenomes, but the abundance of the A subgenome was higher than that of the B and C subgenomes (Supplementary Fig. 1). Collinearity analysis of R2R3-MYBs in six *Brassica species* showed that the collinearity between A and C subgenomes was better than that between B and A or C subgenomes (Fig. 2), which was consistent that their genomic relationships [38]. Therefore, the results offered a useful framework for future research to understand the evolution of the R2R3-MYB gene family.

### The expression level of R2R3-MYBs related to anthocyanin biosynthesis

*Brassica* species present color variations caused by the accumulation of anthocyanins in tissues or organs, such as leaves, stems, flowers, siliques and seeds, from *B. campestris* L. var. *purpuraria* L. H. Bailey, purple cabbage, purple cauliflower, purple *B. juncea*, purple rapeseed. In our study, we analyzed the transcriptional profiles of co-differentially expressed R2R3-MYBs for green and purple leaves in five *Brassica* crops (except *B. nigra*), and a totally of 41 R2R3-MYBs were differentially expressed (Table 2). Of these, eight co-DE R2R3-MYBs were found in five *Brassica* crops (Fig. 3), and were homologous genes of *MYB75*/*MYB90*/*MYB113*/*MYB114* in *Arabidopsis*, and were the subfamily 6 of R2R3-MYB [14, 43]. The co-expression analysis of anthocyanin-related genes and R2R3-MYBs found that *MYB90* have a strong interaction with *TT8* to regulate the structural genes *F3H*, *LDOX, ANS* and *UF3GT* at the same time (Fig. 4). Interestingly, the anthocyanin biosynthesis repressor *CPC* and *MYBL2* were also co-expressed with *TT8*. In this research, comprehensive transcriptome results, phenotypic data and qRT-PCR analysis showed that these six DE R2R3-MYBs are the important regulation genes involved in anthocyanin biosynthesis processes (Fig. 5).

**Fig. 6:**
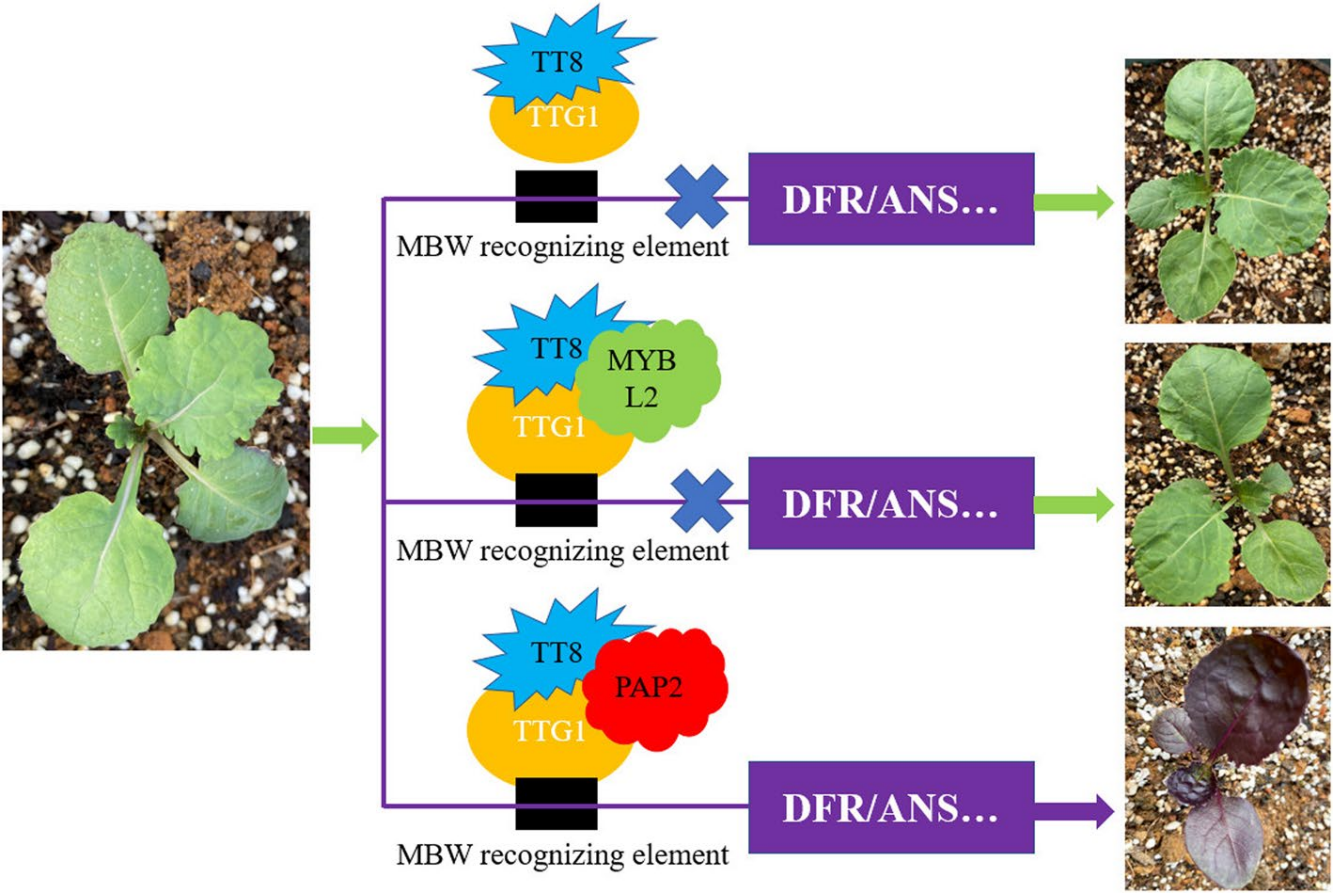
Proposed ‘activator-and-repressor’ loop model for the regulation of anthocyanin accumulation in Brassica species. PAP2 acts as the activator by directly and positively regulating the expression of anthocyanin biosynthetic genes with TTG1 and TT8, whereas MYBL2 acts as the repressor by inhibiting the activity of the PAP2-TT8-TTG1 complex. The arrow and error symbol lines indicate activation or repression, respectively

Previous studies have indicated that R2R3-MYB genes play key roles in the anthocyanin biosynthesis in various plants, such as *TT2*, *MYB75*, *MYB90*, *MYB113* and *MYB114* in *Arabidopsis* ([52]), *Ruby1* and *Ruby2* in citrus (Butelli et al., 2012), *AcMYB123* in kiwifruit [46, 47], *PpMYB10.1* in peaches [35], *MdMYB10* and *MdMYB110a* in Apple [6, 16], and *VvMYBA1* and *VvMYBA2* in grapes [44]. In *Brassica*, previous studies have shown that the sequence variation of the R2R3-MYB transcription factor in the promoter region or the gene intron region to drive its active expression is the main reason for the cumulative variation of *Brassica* anthocyanins. Different types of transposon insertions or sequence variations in the *BoMYB2* promoter in purple *B. oleracea* drove its expression to increase significantly, resulting in different color phenotypes in different tissues or organs of different subspecies [53]. The activated expression of the *BrMYB2* gene in purple leaf Chinese cabbage and the *BjPur* gene in purple leaf *B. juncea* was due to the deletion of the first large intron in the intergenic region [21, 22]. In *B. napus*, *BnaPAP2.A7* was previously found to be a key transcription factor regulating the synthesis of anthocyanins in the leaves [8]. Interestingly, *BoMYB2*, *BrMYB2*, *BjPur*, and *BnaPAP2.A7* were homologous of *Bo6g100940*, *BraA07g032100.3 C*, *BjuB05g10740S* and *BnaA07T0287000ZS*, which was highly consistent with our results. In addition, our results indicated that *MYB90* was the key transcription factor that combined with *TT8* transcription factor and TTG1 protein to form a MBW transcription regulatory complex to regulate the synthesis of anthocyanins in *Brassica* crops, when *MYB90* was not expressed or *MYBL2* inhibited *MYB90* expression by competing with *TT8* and TTG1, *Brassica* crops failed to complete the synthesis and accumulation of anthocyanins (Fig. 6).

In addition, we collected 144 seed coats RNA-seq data at different developmental stages of U’s Triangle six *Brassica* species [18]. Interestingly, co-DE R2R3-MYBs were found that 25 co-DE R2R3-MYBs in the groups UO and Torpedo of six *Brassica* species, but there was no co-differentially expressed R2R3-MYB gene in seed coats and leaves. The analysis of differentially expressed genes related to anthocyanin biosynthesis pathway found that there were many co-differentially expressed structural genes and transcription factors in all six *Brassica* species, such as *DFR*, *LDOX*, *TT2*, *MYB5*, *EGL3* and so on, however, *PAP1*/*PAP2*/*MYB113*/*MYB114* were not differentially expressed in these materials. Comparing the leaf and seed coat transcriptome analysis data of *Brassica* species, we found that the purple phenotype material anthocyanin synthesis pathway structural genes were significantly up-regulated in both leaf and seed coat, while the regulated MYB transcription factors were different. The differentially expressed R2R3-MYB transcription factor was *PAP2* in leaves, but mainly *TT2* and *MYB5* in seed coats. Previously, *BrTT8* and *BrMYB5* may be involved in the regulation of anthocyanins in *B.rapa* seed coat [37],

*BjuTT8* may be regulates the seed coat color variation of *B.rapa*, *B.napus*, and *B.juncea* [40], *BnaC.TT2.a* [57] and *BnTT8* [55] regulates the rapeseed coat color variation. In conclusion, among *Brassica* species, the regulatory genes of anthocyanins in leaves and seed coats may be different, and the regulatory mechanisms need to be further explored.

Overall, the present study provided new insights into the roles of novel R2R3-MYB transcription factors in anthocyanin synthesis of *Brassica* species and into anthocyanin transcriptional regulation in plants.

## Supplementary Information

**Additional file 1: Supplementary Fig. 1.** The distribution of R2R3-MYB on the three subgenomic chromosomes of A, B, and C. Different subgenomic chromosomes represented by different colors, and the same crops are marked with the same color.

**Additional file 2: Supplementary Fig. 2.** Predicted cis-elements in 7 co-differentially expressed R2R3-MYBs promoters and gene structure. Promoter sequences (-2,000 bp) of 7 co-differentially expressed R2R3-MYBs were analyzed using PlantCARE. Different shapes and colors represent different elements.

**Additional file 3: Supplementary Fig. 3.** The domains of R2R3-MYB family genes and protein 3D structural models of R2 and R3 MYB repeats.

**Additional file 4: Supplementary Fig. 4.** Six *Brassica* species with 25 co-differentially expressed R2R3-MYBs correspond to *Arabidopsis*.

**Additional file 5: Supplementary Fig. 5.** The expression pattern of MYBL2 of five *Brassica* species green and purple leaves.

**Additional file 6: Supplementary Table S1.** R2R3-MYB in six *Brassica* species.

**Additional file 7: Supplementary Table S2.** The subfamilies of R2R3-MYB.

**Additional file 8: Supplementary Table S3.** Correspondence between 12 cluster tree subfamilies of *Brassica* R2R3_MYB family and 25 cluster tree subfamilies of *Arabidopsis*.

**Additional file 9: Supplementary Table S4.** Correspondence between the DE R2R3_MYBs of *Brassica* species and *Arabidopsis*.

**Additional file 10: Supplementary Table S5.** Differentially expressed genes and expression levels related to anthocyanin synthesis in seed coat.

**Additional file 11: Supplementary Table S6.** Differentially expressed genes and expression levels of R2R3-MYB in seed coat.

**Additional file 12: Supplementary Table S7.** The details of six *Brassica* species with 25 co-differentially expressed R2R3-MYBs in seed coat correspond to *Arabidopsis*.

**Additional file 13: Supplementary Table S8.** The expression data of R2R3-MYB genes in green and purple leaves of five *Brassica* species.

**Additional file 14: Supplementary Table S9.** Comparison of the identification results of *Arabidopsis* R2R3-MYB genes.

**Additional file 15: Supplementary Table S10.** The detail expression pattern of *PAP2* and *MYBL2* of five *Brassica* species green and purple leaves.

## Data Availability

All materials and related data in this study are available upon request. If you need these materials and related data, you can contact Daozong Chen(chendaozong61@163.com).
