# Supplementary figures and images for: Genome-wide identification of R2R3-MYB gene family and association with anthocyanin biosynthesis in Brassica species
Source: BMC Genomics. 2022 Jun 14;23:441. doi: 10.1186/s12864-022-08666-7 (PMC9199147; doi:10.1186/s12864-022-08666-7)

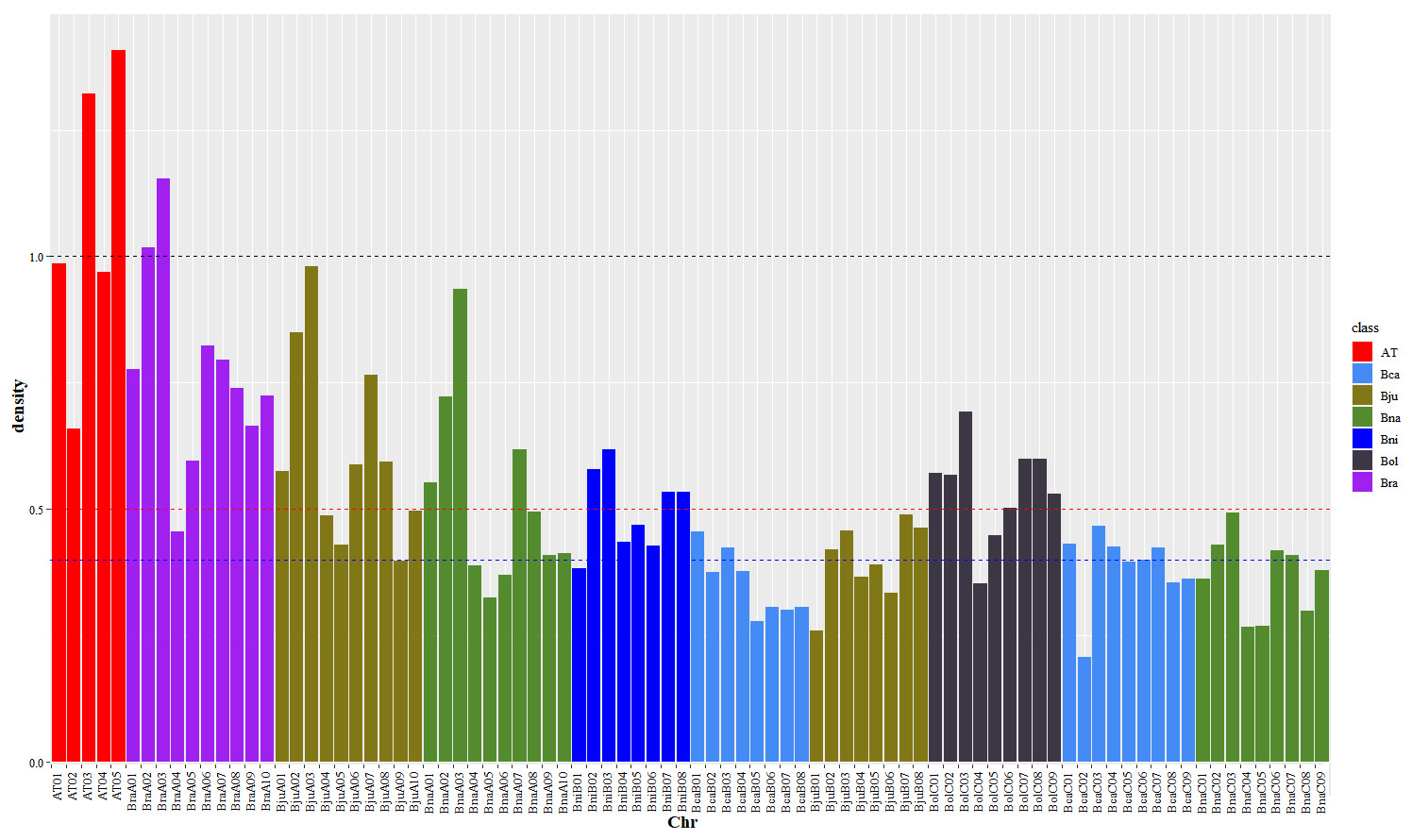

Supplement: Supplementary file 1 — Additional file 1: Supplementary Fig. 1. The distribution of R2R3-MYB on the three subgenomic chromosomes of A, B, and C. Different subgenomic chromosomes represented by different colors, and the same crops are marked with the same color. [file 12864_2022_8666_MOESM1_ESM.jpg]

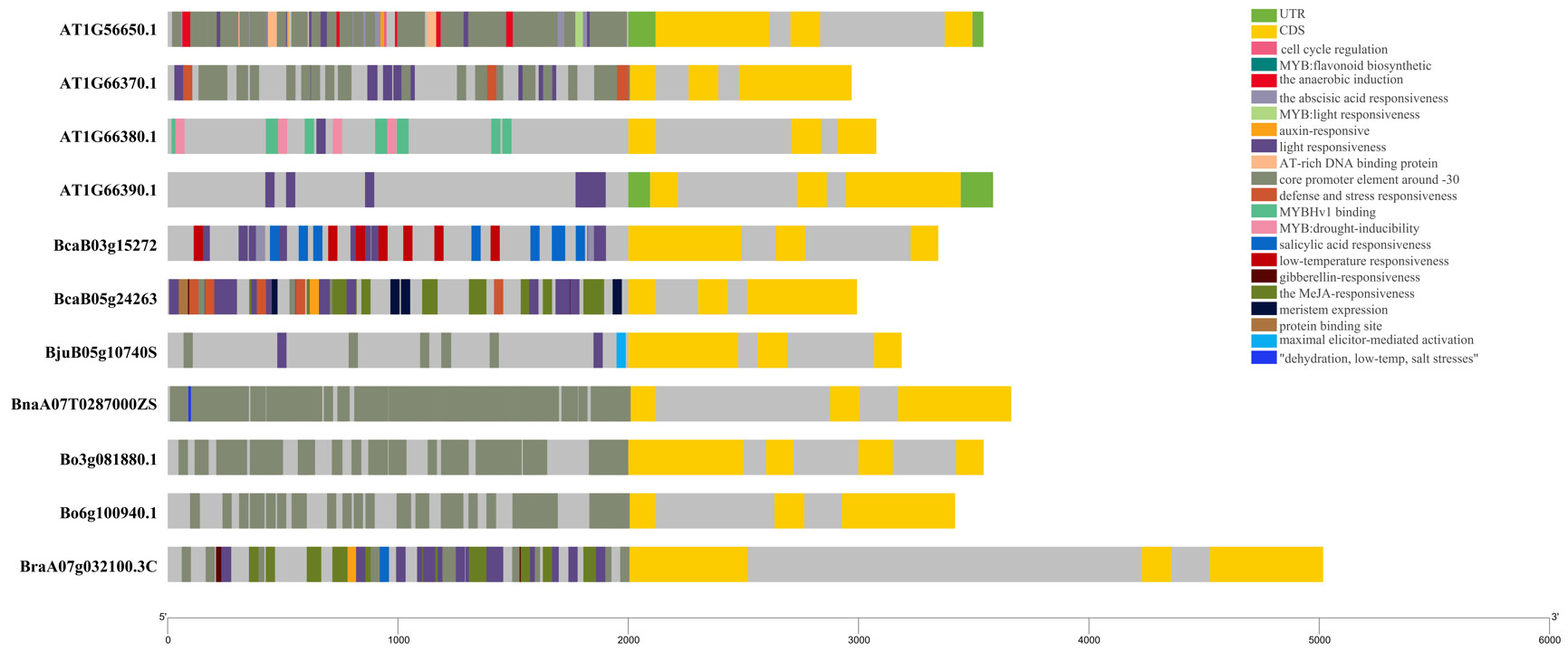

Supplement: Supplementary file 2 — Additional file 2: Supplementary Fig. 2. Predicted cis-elements in 7 co-differentially expressed R2R3-MYBs promoters and gene structure. Promoter sequences (-2,000 bp) of 7 co-differentially expressed R2R3-MYBs were analyzed using PlantCARE. Different shapes and colors represent different elements. [file 12864_2022_8666_MOESM2_ESM.jpg]

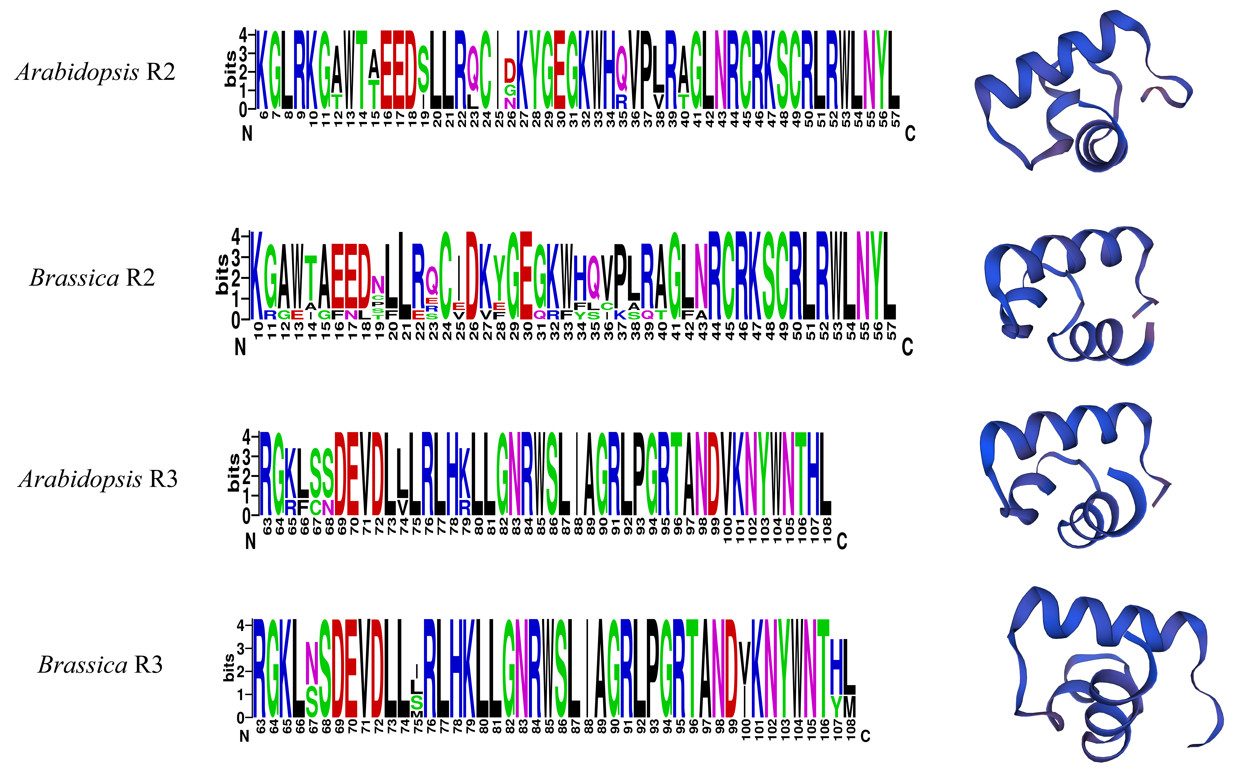

Supplement: Supplementary file 3 — Additional file 3: Supplementary Fig. 3. The domains of R2R3-MYB family genes and protein 3D structural models of R2 and R3 MYB repeats. [file 12864_2022_8666_MOESM3_ESM.jpg]

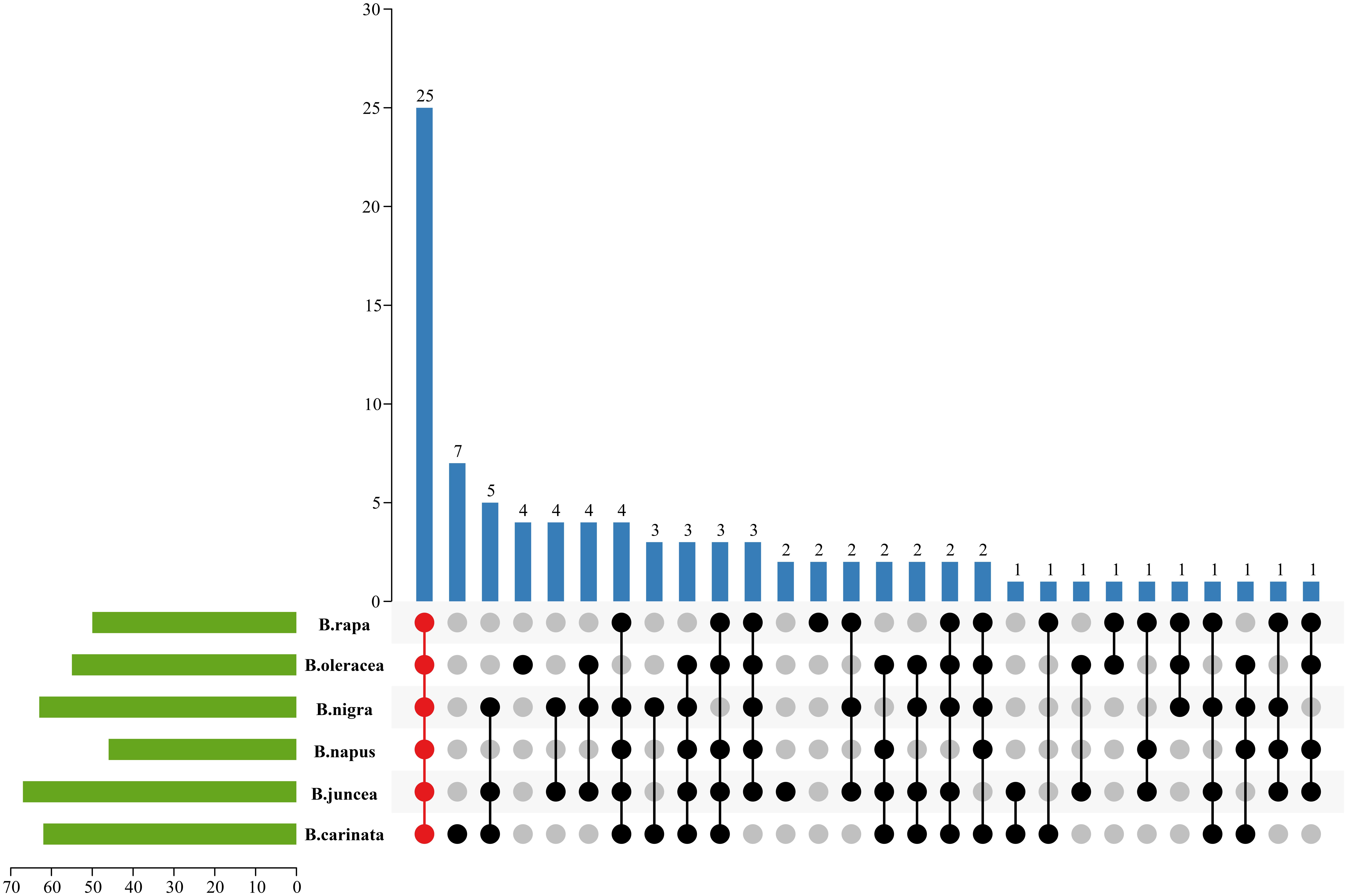

Supplement: Supplementary file 4 — Additional file 4: Supplementary Fig. 4. Six Brassica species with 25 co-differentially expressed R2R3-MYBs correspond to Arabidopsis. [file 12864_2022_8666_MOESM4_ESM.jpg]

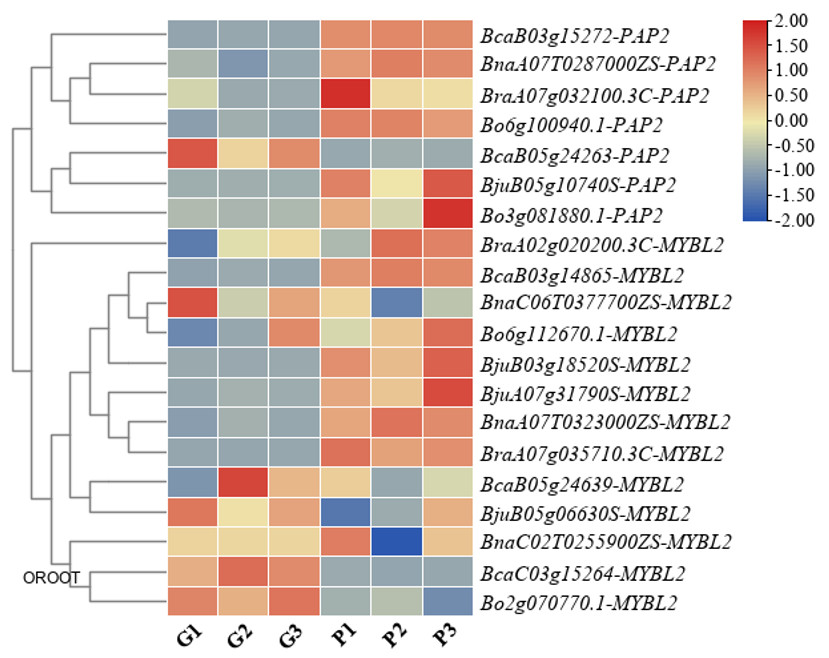

Supplement: Supplementary file 5 — Additional file 5: Supplementary Fig. 5. The expression pattern of MYBL2 of five Brassica species green and purple leaves. [file 12864_2022_8666_MOESM5_ESM.jpg]
