# Supplementary material for: Genome-wide identification of R2R3-MYB gene family and association with anthocyanin biosynthesis in Brassica species
Source: BMC Genomics. 2022 Jun 14;23:441. doi: 10.1186/s12864-022-08666-7 (PMC9199147; doi:10.1186/s12864-022-08666-7)
Supplement: Supplementary file 8 — Additional file 8: Supplementary Table S3. Correspondence between 12 cluster tree subfamilies of Brassica R2R3_MYB family and 25 cluster tree subfamilies of Arabidopsis. [file 12864_2022_8666_MOESM8_ESM.docx]

**Supplementary Table S3.** **Correspondence between 12 cluster tree subfamilies of *Brassica* R2R3_MYB family and 25 cluster tree subfamilies of *Arabidopsis***

| **Brassica** | **T1** | **T2** | **T3** | **T4** | **T5** | **T6** | **T7** | **T8** | **T9** | **T10** | **T11** | **T12** |
| --- | --- | --- | --- | --- | --- | --- | --- | --- | --- | --- | --- | --- |
| **Arabidopsis** | S21 | S22,S23 | S25 |  | S6,S15 | S8,S18,S19,S20 | S13,S16 | S1,S2,S3,S5,S12 | S9,S10,S11,S24 | S14 | S4,S7 | S17 |
