# Supplementary material for: Genome-wide identification of R2R3-MYB gene family and association with anthocyanin biosynthesis in Brassica species
Source: BMC Genomics. 2022 Jun 14;23:441. doi: 10.1186/s12864-022-08666-7 (PMC9199147; doi:10.1186/s12864-022-08666-7)
Supplement: Supplementary file 9 — Additional file 9: Supplementary Table S4. Correspondence between the DE R2R3_MYBs of Brassica species and Arabidopsis. [file 12864_2022_8666_MOESM9_ESM.docx]

**Supplementary Table S4. Correspondence between the DE R2R3_MYBs of *Brassica* species and *Arabidopsis***

| **species** | ***Brassica*** | ***Arabidopsis*** | | | |
| --- | --- | --- | --- | --- | --- |
| ***B.carinata*** | BcaB01g00642 | AT3G50060.1 |  |  |  |
|  | BcaB01g01044 | AT3G46130.1 |  |  |  |
|  | BcaB01g06059 | AT5G07690.1 | AT5G07700.1 |  |  |
|  | BcaB03g15272 | AT1G56650.1 | AT1G66380.1 | AT1G66390.1 |  |
|  | BcaB05g22578 | AT3G46130.1 | AT5G59780.1 |  |  |
|  | BcaB05g24263 | AT1G56650.1 | AT1G66380.1 | AT1G66390.1 |  |
|  | BcaB05g24863 | AT1G74650.1 |  |  |  |
|  | BcaB06g26611 | AT3G24310.1 | AT4G13480.1 |  |  |
|  | BcaC01g01934 | AT3G46130.1 |  |  |  |
|  | BcaC01g02988 | AT2G16720.1 | AT4G34990.1 |  |  |
|  | BcaC01g06550 | AT3G46130.1 | AT5G59780.1 |  |  |
|  | BcaC03g17538 | AT3G28910.1 |  |  |  |
|  | BcaC03g18380 | AT5G67300.1 |  |  |  |
|  | BcaC04g23448 | AT5G61420.1 |  |  |  |
|  | BcaC06g33112 | AT3G50060.1 |  |  |  |
|  | BcaC06g34815 | AT5G67300.1 |  |  |  |
|  | BcaC07g38919 | AT1G18570.1 | AT1G74080.1 | AT5G60890.1 |  |
|  | BcaC07g39472 | AT3G50060.1 |  |  |  |
|  | BcaC08g46983 | AT1G74650.1 |  |  |  |
| ***B.juncea*** | BjuA01g23470S | AT3G50060.1 |  |  |  |
|  | BjuA02g02090S | AT3G47600.1 | AT5G62470.1 |  |  |
|  | BjuA02g21880S | AT1G74650.1 |  |  |  |
|  | BjuA02g37030S | AT5G14340.1 | AT5G16600.1 |  |  |
|  | BjuB02g38830S | AT5G15310.1 |  |  |  |
|  | BjuB02g54940S | AT1G14350.1 | AT2G02820.1 |  |  |
|  | BjuB02g63960S | AT3G50060.1 |  |  |  |
|  | BjuB03g52450S | AT1G18710.1 |  |  |  |
|  | BjuB03g52510S | AT1G18570.1 | AT1G74080.1 | AT5G60890.1 |  |
|  | BjuB05g10740S | AT1G56650.1 | AT1G66380.1 | AT1G66390.1 |  |
|  | BjuB06g16810S | AT1G74650.1 |  |  |  |
|  | BjuB06g57660S | AT2G47460.1 | AT3G62610.1 |  |  |
|  | BjuB07g31540S | AT5G49330.1 |  |  |  |
|  | BjuB08g58100S | AT5G07690.1 | AT5G07700.1 |  |  |
|  | BjuA04g01030S | AT3G61250.1 |  |  |  |
|  | BjuA05g07070S | AT2G37630.1 |  |  |  |
|  | BjuA06g29840S | AT1G18710.1 |  |  |  |
|  | BjuA09g64530S | AT5G62470.1 |  |  |  |
|  | BjuB03g33970S | AT1G22640.1 | AT3G13540.1 |  |  |
|  | BjuB04g48230S | AT1G08810.1 | AT3G28910.1 |  |  |
|  | BjuA03g03860S | AT4G37260.1 |  |  |  |
|  | BjuA03g28580S | AT3G06490.1 |  |  |  |
|  | BjuA03g36010S | AT2G47460.1 | AT3G62610.1 |  |  |
|  | BjuA05g00170S | AT2G47460.1 |  |  |  |
|  | BjuA09g08570S | AT1G18570.1 |  |  |  |
|  | BjuB02g10760S | AT2G16720.1 | AT4G34990.1 |  |  |
|  | BjuB03g40630S | AT4G05100.1 | AT4G21440.1 | AT4G28110.1 | AT5G54230.1 |
|  | BjuB04g37020S | AT1G09770.1 |  |  |  |
|  | BjuB07g34430S | AT5G61420.1 |  |  |  |
| ***B.napus*** | BnaA01T0373700ZS | AT3G11440.1 |  |  |  |
|  | BnaA01T0418000ZS | AT3G01140.1 |  |  |  |
|  | BnaA02T0065300ZS | AT5G14340.1 | AT5G16600.1 |  |  |
|  | BnaA02T0144200ZS | AT5G52600.1 |  |  |  |
|  | BnaA02T0212400ZS | AT1G74650.1 |  |  |  |
|  | BnaA03T0227800ZS | AT2G47460.1 | AT3G62610.1 |  |  |
|  | BnaA03T0376600ZS | AT1G56650.1 | AT1G66370.1 |  |  |
|  | BnaA05T0062900ZS | AT2G39880.1 |  |  |  |
|  | BnaA06T0093300ZS | AT1G14350.1 |  |  |  |
|  | BnaA07T0285700ZS | AT1G66230.1 |  |  |  |
|  | BnaA07T0287000ZS | AT1G66380.1 | AT1G66390.1 |  |  |
|  | BnaC01T0466600ZS | AT3G11440.1 |  |  |  |
|  | BnaC01T0480900ZS | AT3G09370.1 |  |  |  |
|  | BnaC02T0052100ZS | AT5G12870.1 |  |  |  |
|  | BnaC02T0068100ZS | AT5G15310.1 |  |  |  |
|  | BnaC02T0075300ZS | AT5G14340.1 | AT5G16600.1 |  |  |
|  | BnaC02T0083300ZS | AT5G17800.1 |  |  |  |
|  | BnaC02T0205500ZS | AT1G56650.1 |  |  |  |
|  | BnaC02T0523300ZS | AT1G18570.1 | AT1G74080.1 | AT5G60890.1 |  |
|  | BnaC02T0527500ZS | AT5G61420.1 |  |  |  |
|  | BnaC03T0051600ZS | AT4G00540.1 | AT5G11510.1 |  |  |
|  | BnaC03T0298100ZS | AT1G35515.1 | AT4G09460.1 |  |  |
|  | BnaC03T0669700ZS | AT4G38620.1 |  |  |  |
|  | BnaC05T0114500ZS | AT1G14350.1 | AT2G02820.1 |  |  |
|  | BnaC06T0276600ZS | AT1G74650.1 |  |  |  |
|  | BnaC06T0278200ZS | AT1G74430.1 |  |  |  |
|  | BnaC06T0329100ZS | AT1G56650.1 | AT1G66380.1 | AT1G66390.1 |  |
|  | BnaC06T0407500ZS | AT1G18710.1 | AT1G74430.1 |  |  |
|  | BnaC07T0280300ZS | AT2G02820.1 |  |  |  |
|  | BnaC08T0478700ZS | AT1G16490.1 |  |  |  |
|  | BnaC09T0498300ZS | AT5G16600.1 |  |  |  |
|  | BnaC09T0510400ZS | AT5G15310.1 |  |  |  |
|  | BnaA01T0214800ZS | AT3G50060.1 |  |  |  |
|  | BnaA01T0383000ZS | AT3G09370.1 |  |  |  |
|  | BnaA01T0390700ZS | AT3G06490.1 |  |  |  |
|  | BnaA02T0160600ZS | AT1G56650.1 |  |  |  |
|  | BnaA02T0206500ZS | AT1G73410.1 |  |  |  |
|  | BnaA02T0368600ZS | AT5G49620.1 |  |  |  |
|  | BnaA03T0062900ZS | AT5G15310.1 |  |  |  |
|  | BnaA03T0250900ZS | AT1G35515.1 | AT4G09460.1 |  |  |
|  | BnaA03T0283300ZS | AT3G01530.1 |  |  |  |
|  | BnaA03T0303900ZS | AT3G06490.1 |  |  |  |
|  | BnaA03T0399200ZS | AT2G16720.1 |  |  |  |
|  | BnaA03T0414300ZS | AT3G50060.1 |  |  |  |
|  | BnaA03T0561500ZS | AT4G37260.1 |  |  |  |
|  | BnaA05T0004800ZS | AT2G47460.1 |  |  |  |
|  | BnaA05T0117400ZS | AT2G32460.1 | AT3G60460.1 |  |  |
|  | BnaA06T0355400ZS | AT1G48000.1 | AT5G49620.1 |  |  |
|  | BnaA06T0451000ZS | AT4G38620.1 |  |  |  |
|  | BnaA07T0069300ZS | AT3G24310.1 | AT4G13480.1 |  |  |
|  | BnaA07T0141400ZS | AT5G67300.1 |  |  |  |
|  | BnaA07T0250700ZS | AT1G74650.1 |  |  |  |
|  | BnaA07T0310500ZS | AT1G69560.1 |  |  |  |
|  | BnaA07T0338500ZS | AT1G73410.1 |  |  |  |
|  | BnaA08T0297800ZS | AT1G08810.1 |  |  |  |
|  | BnaA09T0021000ZS | AT4G01680.1 |  |  |  |
|  | BnaA09T0078800ZS | AT5G62470.1 |  |  |  |
|  | BnaA09T0514500ZS | AT3G09230.1 | AT3G55730.1 |  |  |
|  | BnaA10T0043200ZS | AT1G06180.1 |  |  |  |
|  | BnaA10T0209700ZS | AT5G15310.1 |  |  |  |
|  | BnaC01T0271000ZS | AT3G50060.1 |  |  |  |
|  | BnaC01T0487800ZS | AT3G06490.1 |  |  |  |
|  | BnaC01T0497100ZS | AT3G01140.1 |  |  |  |
|  | BnaC02T0276500ZS | AT1G73410.1 |  |  |  |
|  | BnaC02T0283700ZS | AT1G74650.1 |  |  |  |
|  | BnaC02T0438100ZS | AT1G14350.1 | AT2G02820.1 |  |  |
|  | BnaC02T0472600ZS | AT3G28910.1 |  |  |  |
|  | BnaC02T0494200ZS | AT5G49620.1 |  |  |  |
|  | BnaC02T0523100ZS | AT5G60890.1 |  |  |  |
|  | BnaC02T0553100ZS | AT5G67300.1 |  |  |  |
|  | BnaC03T0072100ZS | AT5G15310.1 |  |  |  |
|  | BnaC03T0340900ZS | AT3G01530.1 |  |  |  |
|  | BnaC03T0554000ZS | AT3G47600.1 | AT5G62470.1 |  |  |
|  | BnaC03T0689100ZS | AT4G37260.1 |  |  |  |
|  | BnaC04T0005700ZS | AT2G47460.1 |  |  |  |
|  | BnaC04T0069000ZS | AT2G39880.1 |  |  |  |
|  | BnaC04T0146400ZS | AT2G32460.1 | AT3G60460.1 |  |  |
|  | BnaC04T0269900ZS | AT3G61250.1 |  |  |  |
|  | BnaC05T0525300ZS | AT3G06490.1 |  |  |  |
|  | BnaC05T0559400ZS | AT3G01530.1 |  |  |  |
|  | BnaC06T0181400ZS | AT3G53200.1 |  |  |  |
|  | BnaC06T0407800ZS | AT1G74430.1 |  |  |  |
|  | BnaC06T0407900ZS | AT1G74430.1 |  |  |  |
|  | BnaC07T0123100ZS | AT3G23250.1 |  |  |  |
|  | BnaC07T0158200ZS | AT1G25340.1 |  |  |  |
|  | BnaC07T0178800ZS | AT1G22640.1 |  |  |  |
|  | BnaC07T0205300ZS | AT5G67300.1 |  |  |  |
|  | BnaC07T0310800ZS | AT3G27920.1 |  |  |  |
|  | BnaC07T0316500ZS | AT3G28910.1 |  |  |  |
|  | BnaC08T0248000ZS | AT1G18710.1 |  |  |  |
|  | BnaC09T0025700ZS | AT3G27810.1 |  |  |  |
|  | BnaC09T0028400ZS | AT3G28910.1 |  |  |  |
|  | BnaC09T0038300ZS | AT5G49330.1 |  |  |  |
|  | BnaC09T0432300ZS | AT3G46130.1 |  |  |  |
|  | BnaA02T0045300ZS | AT5G12870.1 |  |  |  |
|  | BnaA02T0350200ZS | AT3G28910.1 |  |  |  |
|  | BnaA02T0391300ZS | AT5G60890.1 |  |  |  |
|  | BnaA02T0394700ZS | AT5G61420.1 |  |  |  |
|  | BnaA03T0404300ZS | AT1G18570.1 | AT1G74080.1 | AT5G60890.1 |  |
|  | BnaA03T0547300ZS | AT4G34990.1 |  |  |  |
|  | BnaA06T0129400ZS | AT1G18710.1 |  |  |  |
|  | BnaA08T0186000ZS | AT4G37260.1 |  |  |  |
|  | BnaA09T0051900ZS | AT5G49330.1 |  |  |  |
|  | BnaC06T0409400ZS | AT1G74650.1 |  |  |  |
|  | BnaC07T0376700ZS | AT1G18570.1 | AT1G74080.1 | AT5G60890.1 |  |
| ***B.oleracea*** | Bo1g157620 | AT3G01140.1 |  |  |  |
|  | Bo2g012170 | AT5G14750.1 | AT5G40330.1 |  |  |
|  | Bo2g013970 | AT5G17800.1 |  |  |  |
|  | Bo2g047590 | AT5G52600.1 |  |  |  |
|  | Bo2g161180 | AT1G18570.1 | AT1G74080.1 | AT5G60890.1 |  |
|  | Bo3g010230 | AT5G15310.1 |  |  |  |
|  | Bo4g101460 | AT3G61250.1 |  |  |  |
|  | Bo5g009290 | AT1G08810.1 |  |  |  |
|  | Bo5g025730 | AT1G18710.1 | AT1G74430.1 |  |  |
|  | Bo5g025790 | AT1G18710.1 |  |  |  |
|  | Bo5g043390 | AT1G25340.1 |  |  |  |
|  | Bo6g100940 | AT1G56650.1 | AT1G66380.1 | AT1G66390.1 |  |
|  | Bo7g098110 | AT1G18570.1 | AT1G74080.1 | AT5G60890.1 |  |
|  | Bo7g117140 | AT2G16720.1 | AT4G34990.1 |  |  |
|  | Bo7g118450 | AT4G37260.1 |  |  |  |
|  | Bo8g058040 | AT1G08810.1 |  |  |  |
|  | Bo8g068030 | AT1G18710.1 |  |  |  |
|  | Bo8g104210 | AT1G18710.1 |  |  |  |
|  | Bo9g008700 | AT3G28910.1 |  |  |  |
|  | Bo1g040300 | AT4G25560.1 |  |  |  |
|  | Bo2g010970 | AT5G12870.1 |  |  |  |
|  | Bo2g161590 | AT5G61420.1 |  |  |  |
|  | Bo3g081700 | AT3G23250.1 |  |  |  |
|  | Bo3g081880 | AT1G56650.1 | AT1G66370.1 |  |  |
|  | Bo3g162580 | AT4G21440.1 | AT4G28110.1 |  |  |
|  | Bo6g122640 | AT1G79180.1 |  |  |  |
|  | Bo7g033260 | AT3G24310.1 | AT4G13480.1 |  |  |
|  | Bo7g040780 | AT3G23250.1 |  |  |  |
|  | Bo7g092680 | AT5G49330.1 |  |  |  |
|  | Bo9g172290 | AT5G10280.1 |  |  |  |
| ***B.rapa*** | BraA02g042740.3C | AT5G60890.1 |  |  |  |
|  | BraA03g043830.3C | AT5G60890.1 |  |  |  |
|  | BraA03g044380.3C | AT5G61420.1 |  |  |  |
|  | BraA07g032100.3C | AT1G66380.1 | AT1G66390.1 |  |  |
|  | BraA10g004890.3C | AT1G06180.1 |  |  |  |
|  | BraA01g029810.3C | AT3G24310.1 |  |  |  |
|  | BraA01g039610.3C | AT3G11440.1 |  |  |  |
|  | BraA02g010710.3C | AT3G46130.1 |  |  |  |
|  | BraA02g043710.3C | AT3G47600.1 | AT5G62470.1 |  |  |
|  | BraA03g030470.3C | AT3G01530.1 |  |  |  |
|  | BraA03g040840.3C | AT1G56650.1 | AT1G66370.1 |  |  |
|  | BraA07g008760.3C | AT3G24310.1 | AT4G13480.1 |  |  |
|  | BraA08g012590.3C | AT4G17785.1 |  |  |  |
|  | BraA09g005460.3C | AT5G26660.1 |  |  |  |
